# Association between systemic immune-inflammation index and central obesity in pediatric populations: a cross-sectional and cohort study

**DOI:** 10.3389/fimmu.2025.1546612

**Published:** 2025-02-19

**Authors:** Qian Zhang, Bingxuan Kong, Zhiyu Zhou, Fangqu Liu, Erya Wen, Bingliang Lin, Peng Xuan, Wenlong Lu, Zhe Su, Yanyan Li, Yuhan Tang, Jingfan Xiong, Ping Yao, Yan Li

**Affiliations:** ^1^ Department of Child and Adolescent Chronic Disease Prevention and Control, Shenzhen Center for Chronic Disease Control, Shenzhen, Guangdong, China; ^2^ Department of Nutrition and Food Hygiene, Hubei Key Laboratory of Food Nutrition and Safety, School of Public Health, Tongji Medical College, Huazhong University of Science and Technology, Wuhan, Hubei, China; ^3^ Department of Endocrinology, Shenzhen Children’s Hospital, Shenzhen, China

**Keywords:** immune-inflammation index (SII), central obesity, children and adolescents, cohort study, inflammation

## Abstract

**Background:**

Central obesity in children represents a significant public health concern due to its strong association with an elevated risk of metabolic and cardiovascular disorders. The systemic immune inflammation index (SII) has been implicated in the pathophysiology of obesity-related chronic inflammation. Despite its potential relevance, the specific relationship between central obesity and SII in the pediatric population remains insufficiently explored. The objective of this study was to examine the relationship between SII and central obesity, with a particular focus on the potential of SII as a predictor of central obesity and a means of preventing obesity at an early stage of life.

**Methods:**

Waist-to-height ratio (WHtR), subcutaneous fat, and visceral fat were employed as obesity proxies. Central obesity was defined according to WHtR with a cutoff value of 0.46. The implications of SII on central obesity were examined in a sample of 4,730 individuals in 2021 and validated through a prospective study involving 1,425 subjects in 2023. Cross-sectional associations between SII and central obesity were examined using binomial logistic regression models and generalized linear models. The restricted cubic spline regression was used to explore the non-linear relationship between SII and obesity indicators. In a prospective study, we employed a modified Poisson regression model to investigate the potential causal relationship between SII and central obesity.

**Results:**

Cross-sectionally, adolescents in the highest quartile of SII levels exhibited the greatest risk for central obesity(OR=3.07, 95% CI:2.45~3.87) when compared to those in the lowest quartile. Subgroup analyses showed that higher SII was associated with central obesity. Longitudinally, individuals in the highest SII quartile were found to have the highest risk of developing central obesity (RR=1.83, 95% CI:1.18~2.83) over time.

## Introduction

Obesity has emerged as one of the most prevalent chronic illnesses among children. Studies have shown that the prevalence of overweight and obesity among children and adolescents increased the risk of multiple health problems in adulthood, such as cardiovascular disease, type 2 diabetes, and various cancers, and even increases the risk of all-cause mortality in children (1). This rising prevalence not only has long-term health consequences but also creates a huge economic burden. According to the 2019 Global Burden of Disease Study, obesity significantly increases the global burden of metabolic diseases, measured by disability-adjusted life years and premature death (2, 3). The economic and social costs associated with childhood obesity are significant, and the financial burden on health systems is consequently increased (4). Accordingly, it is imperative to implement strategies aimed at preventing the onset of overweight and obesity in early life, given the long-term persistence of overweight and obesity and their detrimental impact on physical health.

It is crucial to identify a simple, accurate, and scientifically robust measure of obesity in children and adolescents. While Body Mass Index (BMI) is commonly used, it has notable limitations. BMI does not differentiate between fat and lean body mass, nor does it account for the distribution of body fat—factors that are key to understanding health risks (5). The waist-to-height ratio (WHtR) is a validated, cost-effective, and practical alternative and it is also applicable to the pediatric population (6). It better captures abdominal adiposity, strongly correlates with cardiovascular risk factors, and independently predicts conditions such as type 2 diabetes, dyslipidemia, hypertension, and coronary artery disease (7–10). WHtR is thus a superior tool for assessing obesity and associated health risks in adolescents. Therefore, this study used WHtR as an indicator of central adiposity and used a cut-off value of 0.46 to explore its relationship with Immune-Inflammatory Index(SII).

Systemic inflammation plays a critical role in the pathogenesis of chronic diseases, including obesity, which is characterized by chronic, low-grade inflammation (11, 12). Adiposity disrupts metabolic homeostasis through elevated inflammatory mediators, leading to an imbalance between pro- and anti-inflammatory immune cells (13). Central obesity, in particular, exacerbates this inflammatory state and is linked to increased cardiometabolic risk, including metabolic syndrome (MetS), as indicated by biomarkers and high waist circumference (WC) (11). The Systemic Immune-Inflammatory Index (SII), a novel inflammatory marker initially used in tumor prognosis (14), has shown predictive value in inflammatory diseases (15). Significantly associated with C-reactive protein (CRP) (16), the SII offers a cost-effective and straightforward method for assessing systemic inflammation. Unlike traditional markers such as the neutrophil-to-lymphocyte ratio (NLR) (17) and platelet-to-lymphocyte ratio (PLR) (18), SII provides a more comprehensive reflection of inflammatory status and is particularly advantageous for evaluating inflammation in children and adolescents (19, 20).

Recent findings suggest a potential link between SII and obesity-related inflammation, with studies showing associations between SII, high WC, and cardiometabolic risk biomarkers (11). However, the role of SII in evaluating obesity-induced inflammation remains underexplored. Investigating this relationship could offer new insights into preventing obesity-related comorbidities.

Therefore, we aimed to explore the relationship between inflammatory status, as measured by SII, and central obesity in children and adolescents, investigating the role of SII in the onset and progression of these conditions by discovering it in a cross-sectional framework and subsequently validating it through cohort analysis.

## Methods

### Study design and participants

Participants were recruited from the Evaluation and Monitoring of School-based Nutrition and Growth in Shenzhen (EMSNGS), an ongoing longitudinal study in Shenzhen, China. The baseline survey was completed in 2021, comprising 5,031 children aged 6-17 years, selected through a multi-stage stratified cluster sampling method. A follow-up was conducted in 2023 with 2,154 students from grades 1 to 5. Informed consent was obtained from all participants and their guardians prior to inclusion in the study. The protocol was approved by the ethics review committee of the Shenzhen Center for Chronic Disease Control (SZCCC-2021-037-01-PJ) and registered at the China Clinical Trials Registry (ChiCTR2100051722).

In this research, to figure out the association between SII and central obesity, discovered and validated study were conducted. Participants were selectively excluded according to defined exclusion criteria. Subjects who older than 18 years of age (n=17), who lacked of waist circumference and height data (n=15), who had incomplete inflammatory marker measurements (n=37), who did not have complete covariate information (n=198), or who develop serious illnesses were excluded (n=34). In a further cohort study, we removed people who already had central obesity at baseline. Finally, in our study, 4,730 participants met the inclusion criteria for the cross-sectional analysis, while 1,425 participants were included in the follow-up study after excluding those who were overweight or obese at baseline (**Supplementary Figure 1**).

### Outcome assessment

The presence of central obesity is determined by a WHtR greater than 0.46, which is considered a threshold value for this condition (6). Height was measured using standardized equipment (SH-200, Shanghe, Zhengzhou, China) to the nearest 0.1 cm. Waist circumference was assessed with a girth scale (Hoechstmass, National Institute of Sports Science, China), accurate to 0.1 cm and capable of measuring up to 200 cm. Both height and waist measurements were conducted by trained medical professionals. For height measurement, subjects were instructed to remove footwear and socks, stand upright with knees together, and maintain a neutral head position. Waist circumference was measured at the end of a quiet exhalation, with each measurement taken twice and the mean value recorded, The distribution of body fat can be more accurately assessed by measuring both subcutaneous and visceral fat, using a body composition analyzer (BAS-H, Beijing Sihai Huachen Technology Ltd Company, China).

### Laboratory measurement

Venous blood samples were collected from the subjects in the morning.Venous blood samples were collected from the subjects under investigation and placed in 4ml vacuum EDTA-K2 anticoagulant tubes and subsequently subjected to routine blood tests conducted by laboratory clinical laboratory staff using the automated hematology analyzer (BC-7500CRP). The SII is calculated as follows: SII = Platelet Count × (Neutrophil Count/Lymphocyte Count).

### Covariates

Confounding factors associated with SII and WHtR were analyzed based on previous studies. All covariates on sociodemographics and lifestyle were obtained from structured questionnaires, with the collection of information on the questionnaires being completed by both parents and students for students in years 1-3, and by the students themselves in the remaining grades. In the present study, gender, age, nation (Han and others), drinking status, smoking status (never smoked and drank alcohol were divided as non-smokers and non-drinkers), income (annual income <120,000CNY was considered low income; 120,000~499,999CNY was considered middle income, and >500,000CNY was considered high income), screen time (<2h/d considered as decent), outdoor activities (>1h/d considered as sufficient), exercise time (one hour of moderate to high intensity activity per day is judged to be sufficient.), parents’ education level (whichever parent was educated more than 15 years), and parents’ BMI (calculated by parent’s self-reported height and weight) were considered as covariates.

### Statistical analysis

Numerical variables were described using medians and interquartile ranges (25th and 75th percentiles), after assessing for normality using the Kolmogorov-Smirnov test. Non-parametric comparisons between the two groups were conducted using the Mann–Whitney U test. Categorical variables were expressed as percentages and analyzed for group differences using Chi-squared tests.

The SII was divided by 100 as the original data to improve statistical efficiency. Furthermore, we divided SII values into four groups according to the quartile and assessed the linear trend. In cross-sectional analyses, the relationship between SII and central obesity was investigated through the use of binary logistic regression, while the correlation between SII and body fat distribution was examined through the application of generalized linear models. Furthermore, stratified analyses were conducted based on key characteristics such as gender, age, income, smoking status, parental education, and parental BMI. A restricted cubic spline (RCS) regression was used to investigate the dose-response relationship between SII and obesity, with multivariable adjustments as mentioned above. In the prospective study, an additional investigation was conducted to ascertain the causal relationship between SII and central obesity, employing modified Poisson regression (21). Three regression models were created to test the stability of the results by adjusting the confounding factors stepwise. Model 1 was unadjusted. Model 2 was adjusted for gender, age, nation. Model 3 was further adjusted for drinking status, smoking status, income, screen time, outdoor activities, exercise time, the educational level of parents and parents’BMI.

All statistical analyses were conducted using SPSS (version 26.0; IBM Corp.) and R (Version 4.3.1 for Windows). A *P* value of less than 0.05 was considered statistically significant (two-tailed).

## Results

### Participants characteristics

The baseline characteristics of the study population are detailed in **Table 1**. The survey included 4,730 participants, with a prevalence of central obesity among adolescents at 20.27%. The average age was 11.83 years for participants with normal weight and 12.27 years for those classified as central obesity. Notably, compared to the normal-weight counterparts, obese individuals were more likely to consume alcohol, and have parents with a predisposition towards obesity.

**Table 1:** Baseline charactersitics of participants from EMSNGS according to WHtR ^1^.

	Total	Normal (n=3771)	Obesity (n=959)	*P-value* ^2^
Age (years)	11.91 (9.16,14.71)	11.83 (9.02,14.62)	12.27 (9.74,15.17)	<0.001
Gender (%)				<0.001
Boy	2622 (55.43)	1998 (52.98)	624 (65.07)	
Girl	2108 (44.57)	1773 (47.02)	335 (34.93)	
Drink (%)				0.009
Yes	882 (18.64)	675 (17.89)	207 (21.58)	
No	3848 (81.35)	3096 (82.10)	752 (78.41)	
Smoke (%)				0.519
Yes	91 (1.92)	75 (1.99)	16 (1.67)	
No	4639 (98.08)	3696 (98.01)	943 (98.33)	
Screen time (%)				0.055
≤2h/d	3902 (82.49)	3131 (83.03)	771 (80.40)	
>2h/d	828 (17.51)	3446 (16.97)	188 (19.60)	
Outdoor activity time (%)				0.657
>1h/d	412 (8.71)	325 (8.62)	87 (9.07)	
≤1h/d	4318 (91.29)	3446 (91.38)	872 (90.93)	
Exercise time (%)				0.950
>1h/d	1249 (26.41)	995 (26.39)	254 (26.49)	
≤1h/d	3481 (73.59)	2776 (73.61)	705 (73.51)	
BMI of Father	24.21 (22.39,25.96)	23.94 (22.13,25.83)	24.80 (23.05,26.73)	<0.001
BMI of Mother	21.48 (19.98,23.31)	21.34 (19.78,23.01)	22.48 (20.81,24.41)	<0.001
Education of Parents (%)				0.824
less than15 years of education	1446 (30.57)	1150 (30.50)	296 (30.87)	
more than 15 years of education	3284 (69.43)	2621 (69.50)	663 (69.13)	
Nation (%)				0.425
Han	4544 (96.07)	3627 (96.18)	917 (95.62)	
Others	186 (3.93)	144 (3.82)	42 (4.4)	
Income (%)				0.069
<120,000 CNY	1233 (26.07)	1003 (26.60)	230 (23.98)	
120,000-500,000 CNY	2817 (59.55)	2245 (59.53)	572 (59.65)	
≥500,000 CNY	680 (14.38)	523 (13.87)	157 (16.37)	

BMI, body mass index; CNY, China Yuna.

^1^Data are expressed as median (P25,P75) or frequency (Percentage).

^2^Chi-square, Mann-Whitney U tests were used.

As demonstrated in **Supplementary Table 1**, the visceral fat area and percentage of subcutaneous fat were markedly elevated in the abdominal obesity group relative to the normal group. Moreover, the total leukocyte count, lymphocyte count, neutrophil count, and platelet count were significantly elevated in the central obesity group.

### Cross-sectional study

As depicted in **Table 2**, SII was remarkably associated with abdominal obesity. Specifically, compared with the first quantile of SII, the fourth quantile was associated with a higher risk of central obesity (OR=2.82, 95% CI: 2.26~3.52) in unadjusted model. In the crude model, comparing with lowest quantile, the ORs for the highest was 3.01(95% CI: 2.41~3.77). In fully adjusted model, comparing with lowest quantile, the ORs for central obesity were 1.86 (95% CI: 1.47~2.36), 2.46 (95% CI: 1.96~3.11) and 3.07 (95% CI: 2.45~3.87) for successive quintiles of SII, respectively. When SII was employed as a continuous variable, the ORs resulting from a 100-unit change were 1.12(95% CI: 1.10~1.15), 1.13(95% CI: 1.10,1.17) and 1.13(95% CI: 1.10,1.17) in the three models, respectively.

**Table 2:** Association between SII and central obesity in the cross-sectional study ^1^ (N=4730).

	Quartiles of SII, ×10^9^/L				*P* for trend ^2^	Per 100×10^9^/L increasead
	Q1 (n=1296)	Q2 (n=1299)	Q3 (n=1075)	Q4 (n=1060)		
	OR (95%CI)					OR (95%CI)
Range of SII (10^9^/L)	40.99~305.77	305.84~412.94	412.98~556.99	557.07~3056.97		
Numbers of obesity (%)	137 (11.58)	227 (19.27)	276 (23.27)	319 (26.94)		
Model1	1.00 (Ref.)	1.82 (1.45~2.30)***	2.32 (1.86~2.90)***	2.82 (2.26~3.52)***	<0.001	1.12 (1.10~1.15)***
Model2	1.00 (Ref.)	1.83 (1.46~2.31)***	2.42 (1.94~3.04)***	3.01 (2.41~3.77)***	<0.001	1.13 (1.10~1.17)***
Model3	1.00 (Ref.)	1.86 (1.47~2.36)***	2.46 (1.96~3.11)***	3.07 (2.45~3.87)***	<0.001	1.13 (1.10~1.17)***

OR, odds ration; CI, confidence interval; Q, quartile; SII, systemic immune-inflammation index.

^1^Binary logistic regression was used. Model1: unadjust model. Model2: adjust for gender, age, nation. Model3: adjust for gender, age, nation, drinking status, smoking status, income, screen time, outdoor activities, exercise time, the educational level of parents, and parents’ BMI.

^2^The *P* value for the trend was assessed by assigning the median value for each quartile of SII as a continuous variable.

*P<0.05; **P<0.01; ***P<0.001.

**Figure 1:**
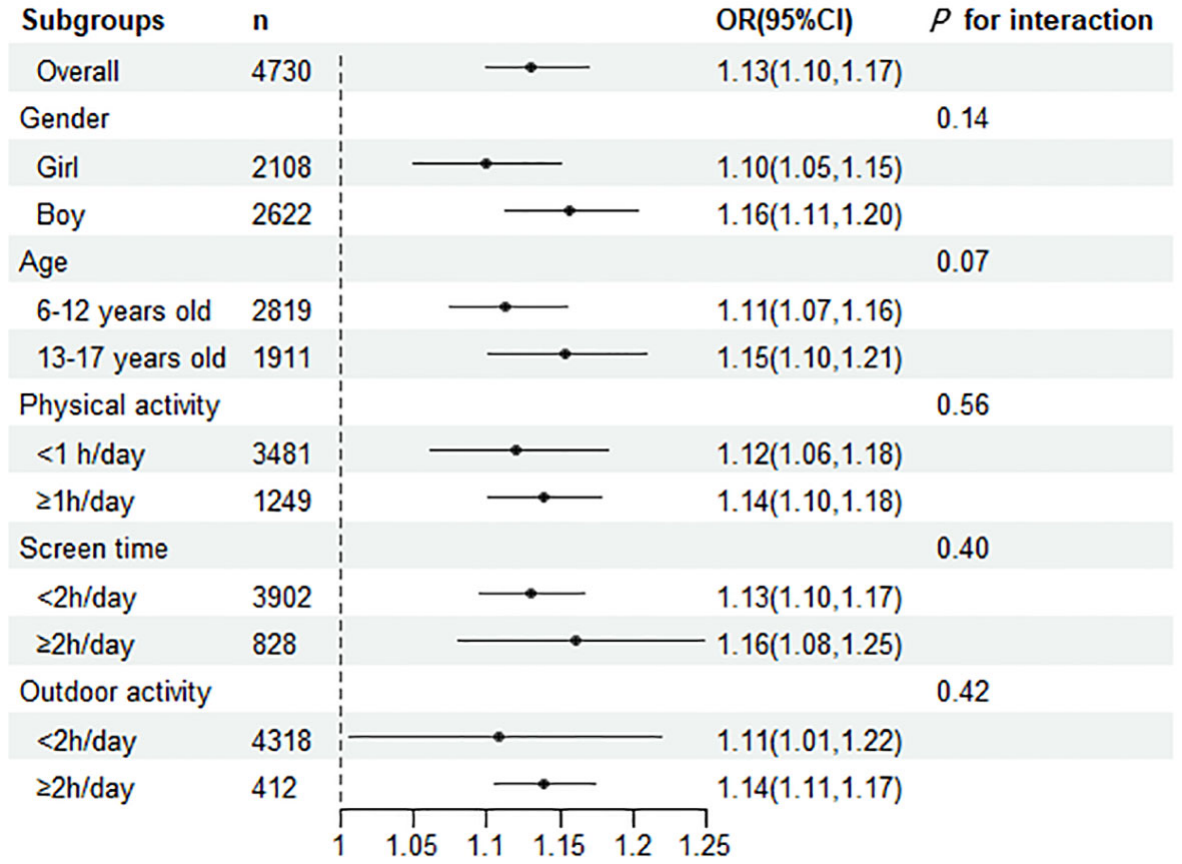
Subgroup analyses were performed for the risk of central obesity per 100 units of change in the SII. The OR represented by fully adjusted model.

We stratified the results according to age, gender, screen time, time spent outdoors, and time spent in physical activity, and the results were consistent across all strata, suggesting that children with higher levels of SII are at higher risk of developing central obesity. Besides, in specific subgroups(such as girls, olders), a higher SII was more strongly correlated with a higher prevalence of central obesity. However, we found no interaction in any of subgroups (**Figure 1**).

**Figure 2:**
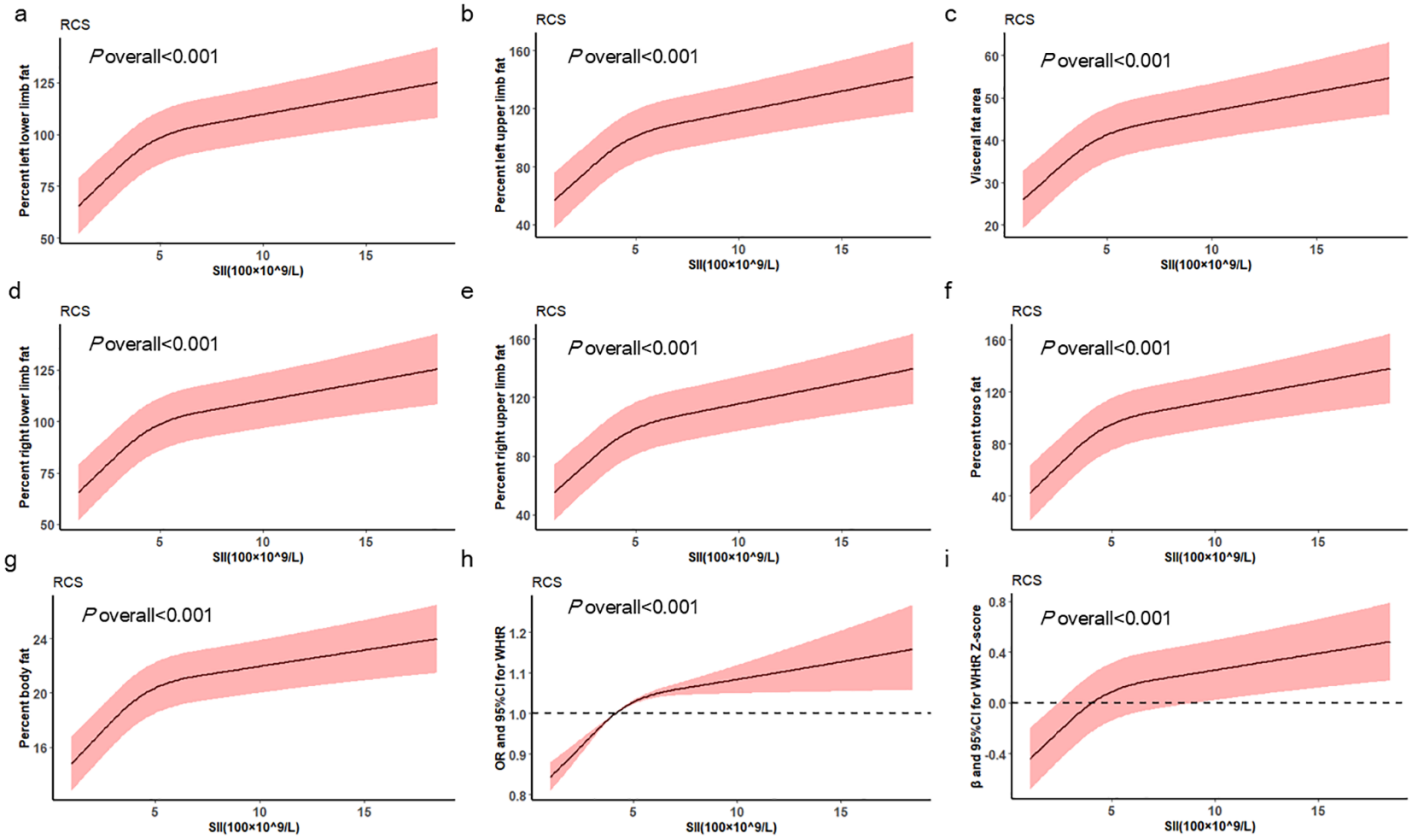
RCS Curves of SII and central obesity and body fat in the cross-sectional study.

As illustrated in **Supplementary Table 2**, after adjusting for multiple confounding variables, there was a significant positive correlation between SII and body fat percentage(*P <*0.001). RCS illustrates the non-linear relationship between SII and body fat percentage (**Figure 2**).

### Cohort study

The cohort analysis excluded individuals diagnosed with obesity at baseline, focusing on the incidence of obesity during the follow-up period. Of the 1,425 participants included in the cohort study, the overweight and obesity rate was 9.82%.

It can be observed that **Table 3** showed a significant correlation between SII and central obesity in the longitudinal analysis. In the unadjusted model, the highest SII group had a higher prevalence of central obesity(RR=1.82, 95% CI: 1.16~2.84). The association remained after adjusting for age, gender and nation. The fully adjusted model indicated that individuals in the fourth quartile of SII were more prone to developing peripheral obesity(RR=1.83, 95% CI: 1.18~2.83), as defined by WHtR, compared to those in the first quartile. Similarly, the prevalence of central obesity also demonstrated a notable correlation with each 100-unit increase in SII. Body fat percentage(*β*=0.15, 95% CI: 0.02~0.29), torso fat percentage (*β*=1.76, 95%CI: 0.53~2.98), limb fat percentage, and visceral fat area (*β*=0.39, 95%CI: 0.06~0.62) all increased significantly with each 100-unit increase in SII (**Supplementary Table 3**).

**Table 3:** Association between SII and central obesity in the cohort study (N=1425) ^1^.

	Quartiles of SII, ×10^9^/L				*P* for trend^2^	Per 100×10^9^/L increasead	ΔSII ^3^
	Q1 (n=357)	Q2 (n=356)	Q3 (n=356)	Q4 (n=356)			
	RR (95%CI)					RR (95%CI)	
Range of SII (10^9^/L) in 2021	40.99~283.20	283.62~388.23	388.43~526.44	528.00~2574.48			
Range of SII (10^9^/L) in 2023	41.42~282.14	282.28~381.66	382.46~518.49	518.50~2278.89			
Numbers of obesity (%)	27 (7.56)	30 (8.43)	34 (9.55)	49 (13.76)			
Model1	1.00 (Ref.)	1.11 (0.68~1.83)	1.26 (0.78~2.05)	1.82 (1.16~2.84)**	<0.001	1.08 (1.04~1.13)**	1.77 (1.23~2.53)**
Model2	1.00 (Ref.)	1.12 (0.68~1.84)	1.27 (0.79~2.04)	1.85 (1.19~2.88)**	0.004	1.09 (1.04~1.13)***	1.78 (1.24~2.54)**
Model3	1.00 (Ref.)	1.13 (0.69~1.86)	1.28 (0.75~2.20)	1.83 (1.18~2.83)**	0.005	1.09 (1.04~1.13)***	1.74 (1.21~2.49)**

RR, relative risk; CI, confidence interval; Q, quartile; SII, systemic immune-inflammation index.

^1^Modified Poisson regression was used. Model1: unadjust model. Model2: adjust for gender, age, nation. Model3: adjust for gender, age, nation, drinking status, smoking status, income, screen time, outdoor activities, exercise time, the educational level of parents, and parents’ BMI.

^2^The *P* value for the trend was assessed by assigning the median value for each quartile of SII as a continuous variable.

*P<0.05; **P<0.01; ***P<0.001.

^3^This examines the impact of changes in SII from 2021 to 2023 on the incidence of central obesity in children. Using the group with decreased or unchanged SII as the reference, the analysis focuses on the association between increased SII and new-onset central obesity. Baseline SII was adjusted in all models (Model 1, Model 2, and Model 3) to account for its potential influence on the development of central obesity in children.

## Discussion

The present study revealed a correlation between SII and central obesity, with SII exhibiting a positive correlation with abdominal obesity. Interestingly, modified Poisson regression was employed in the cohort study to suggest that increased SII may be a precursor of obesity, challenging the traditional perspective that obesity primarily drives inflammation (16, 22). These results endorse the utility of SII as a marker of central obesity. We also found that an elevated SII was associated with an increased percentage of fat content. To our knowledge, this is the first investigation that systematically explores the relationship between SII and central obesity measured as WHtR among children and adolescents.

Given the link between inflammation and peripheral blood cell changes, there is a growing trend to evaluate the potential value of hematological pro-inflammatory markers in the diagnosis and prognosis of various chronic diseases such as obesity (23, 24). In addition to classical inflammatory biomarkers such as CRP, leukocytes and neutrophils, the most studied whole blood cell indicators include NLR and PLR (25, 26). Such cost-effective markers are easily obtained from routine blood tests and provide important information about the state of systemic inflammation. SII, which integrates neutrophil, lymphocyte, and platelet counts, offers an objective measure of inflammation that surpasses traditional markers like NLR and PLR in the depth of information and accessibility (15, 27). It has proven to be robust across varying physiological conditions, suggesting its reliability as a consistent marker of inflammation (28). While SII’s association with chronic diseases such as tumors and cardiovascular diseases has been well documented (16, 20, 29, 30), its link to obesity is less established.

Our analysis revealed strong positive correlations between SII and central obesity, consistent with other studies (31–34). However, it should be noted that other studies have utilized WC as a marker of central obesity. Another study of US adults demonstrated a significant correlation between SII and obesity, as measured by BMI. However, when central obesity was defined by WC, this correlation was no longer observed (35). This discrepancy may be attributed to the fact that the study population was predominantly comprised of Chinese children and adolescents. Furthermore, there are no established waist circumference determination criteria for children. Studies demonstrated a significant association between WHtR and all components of MetS. Furthermore, WHtR was identified as a superior predictor of dyslipidemia, diabetes and cardiometabolic diseases (36), obesity in childhood period increases the risk of cardiometabolic disease through inflammation and oxidative stress (6). This lends further support to the notion that indicators of abdominal obesity offer a more robust predictive capacity for metabolic risk. Moreover, the findings of the SII in predicting metabolic syndrome in obese children indicate that SII can be utilized as a cardiometabolic risk biomarker, thereby facilitating enhanced routine preventive care and diagnosis of metabolic syndrome in obese children (11). This provides further evidence of SII’s capacity to predict obesity and additionally illustrates its potential clinical utility. Indeed, it has been demonstrated that the percentage of body fat in children and adolescents is associated with inflammation, as indicated by CRP and absolute neutrophil count levels (37). Our utilization cohort corroborated this relationship and demonstrated that inflammation is a significant contributing factor to an increase in body fat percentage.

Our findings suggest an intricate interplay between immune cell homeostasis and obesity. It has been demonstrated that an elevation in SII values is indicative of an increase in neutrophil and platelet counts, as well as an augmentation in the levels of several cytokines (and/or a reduction in lymphocyte counts). A decline in lymphocytes during the inflammatory process can lead to an increase in the production of pro-inflammatory cytokines, which in turn induces oxidative stress, thereby promoting the aggregation of inflammation and facilitating disease progression (38). Elevated neutrophil counts observed in overweight and obese adolescents could indicate early immune response activation, which has been linked to the progression of metabolic disorders (39). The obesity-related inflammation is a consequence of immune-related dysfunction in adipose tissue, which involves the transient infiltration of neutrophils within abdominal fat and their binding to adipocytes (40). These findings underscore the pivotal role of inflammation in the progression of obesity and its metabolic complications.

This study has several notable strengths. Primarily, we were able to identify the role of SII in abdominal obesity as defined by WHtR in a population of children and adolescents. Furthermore, we employed a combination of cross-sectional and cohort studies to comprehensively confirm the relationship between SII and abdominal obesity. Despite these insights, the study has limitations. Firstly, it was not possible to completely eliminate the effect of unmeasured confounders on the outcomes. Secondly, during the course of our analyses, we exclude participants with incomplete data, which may result in a degree of selection bias. Thirdly, the sample was drawn from a single city, which may have introduced bias and limited the generalizability of the findings to child and adolescent populations across the country. Therefore, expanding the study to a broader demographic and increasing the sample size would help validate the robustness of these findings.

## Conclusion

Overall, our research underscores the potential of SII as an effective marker for detecting obesity-related inflammation in pediatric populations, offering new avenues for early intervention and prevention of associated comorbidities.

## Data Availability

The raw data supporting the conclusions of this article will be made available by the authors, without undue reservation.
