# Supplementary material for: Association between systemic immune-inflammation index and central obesity in pediatric populations: a cross-sectional and cohort study
Source: Front Immunol. 2025 Feb 19;16:1546612. doi: 10.3389/fimmu.2025.1546612 (PMC11880016; doi:10.3389/fimmu.2025.1546612)
Supplement: Supplementary file 1 [file DataSheet1.docx]

***Supplementary Material***

**Tables:**

**Supplementary Table 1**  Baseline clinical and biological parameters of participants

**Supplementary Table 2** Association between SII and body fat in the cross-sectional study(N=4686)

**Supplementary Table 3** Association between SII and body fat in the cohort study(N=1423)

**Supplementary Table 1** Baseline clinical and biological parameters of participants ^1^.

|  | Total | Normal  (n=3771) | Obesity  (n=959) | *P-value* ^3^ |
| --- | --- | --- | --- | --- |
| WBC(×10^9^/L) | 6.94(6.02,8.14) | 6.82(5.92,7.95) | 7.43(6.50,8.74) | <0.001 |
| LY(×10^9^/L) | 2.64(2.24,3.10) | 2.63(2.23,3.07) | 2.69(2.30,3.16) | 0.002 |
| NEU(×10^9^/L) | 3.56(2.85,4.45) | 3.46(2.77,4.32) | 3.94(3.23.4.92) | <0.001 |
| PLT(×10^9^/L) | 306.00  (265.75,352.00) | 303.00  (264.00,349.00) | 319.00  (275.00,365.00) | <0.001 |
| SII(×10^9^/L) | 413.46  (305.72,557.17) | 399.44  (294.82, 540.85) | 463.30  ( 360.41,630.15) | <0.001 |
| Visceral fat area(cm^2^) ^2^ | 30.15(17.90,51.80) | 25.00(16.70,40.30) | 72.00(48.00,98.95) | <0.001 |
| Torso fat rate(%)^a^ | 81.00  (32.00,146.83) | 61.20  (22.30,106.75) | 227.00  (178.15,283.20) | <0.001 |
| Fat of left upper limb(%) ^2^ | 105.20  (74.58,149.00) | 93.00  (68.50,121.70) | 212.30  (165.75,278.85) | <0.001 |
| Fat of right upper limb(%) ^2^ | 102.70  (72.40,146.50) | 90.70  (66.40,119.20) | 209.3  (162.95,274.95) | <0.001 |
| Fat of left lower limb(%) ^2^ | 102.40  (75.10,140.35) | 91.20  (70.30,116.15) | 184.00  (150.25,228.60) | <0.001 |
| Fat of right lower limb(%) ^2^ | 102.70  (75.30.140.73) | 91.40  (70.50,116.50) | 184.70  (151.50,229.90) | <0.001 |
| FMP(%) | 18.30(12.80,25.80) | 16.00(11.70,21.70) | 30.40(25.50,34.60) | <0.001 |
| WC | 63.00 (55.70,70.96) | 60.50(54.00,66.00) | 78.00(71.00,84.50) | <0.001 |
| WHtR | 0.41(0.39,0.45) | 0.40(0.38,0.42) | 0.50(0.48,0.53) | <0.001 |

WBC, white blood cell count; LY, lymphocytes; NEU, neutrophils; SII, systemic immune-inflammation index; FMP, fat mass percentage; WC, waist circumstance; WHtR, waist-height ratio.

^1^ Data are expressed as median (P25,P75).

^2^ Data represents the percentage of fat distribution, with a sample size of 4,686.

^3^ Mann-Whitney U tests were used.

**Supplementary Table 2** Association between SII and body fat in the cross-sectional study ^1^(N=4686)_._

|  | Quartiles of SII, ×109/L | | | | *P* for trend^2^ | Per 100×10^9^/L increasead |
| --- | --- | --- | --- | --- | --- | --- |
|  | Q1  (n=1172) | Q2  (n=1171) | Q3  (n=1172) | Q4  (n=1171) |  |  |
|  | *β*(95CI%) | | | |  | *β*(95%CI) |
| Range of SII(10^9^/L) | 40.99~305.97 | 305.98~413.37 | 413.54~557.38 | 557.38~3056.97 |  |  |
| Body fat Percentage | 0(Ref.) | 1.89  (1.27~2.52)*** | 3.43  (2.80~4.05) *** | 4.30  (3.67~4.92)*** | <0.001 | 0.62  (0.52~0.71) *** |
| Visceral fat area | 0(Ref.) | 5.29  (3.17~7.41) *** | 8.81  (6.68~10.93) *** | 13.27  (11.13~15.41)*** | <0.001 | 1.86  (1.55~2.17) *** |
| Torso | 0(Ref.) | 19.19  (12.48~25.89 )*** | 28.47  (22.47~34.48)*** | 36.89  (30.85~42.93) *** | <0.001 | 6.29  (5.30~7.28)*** |
| Percentage of left upper limb | 0(Ref.) | 15.55  (9.56~21.54)*** | 28.47  (22.47~34.48)*** | 36.92  (30.88~42.97)*** | <0.001 | 5.51  (4.63~6.39) *** |
| Percentage of right upper limb | 0(Ref.) | 15.55  (9.56~21.53)*** | 27.95  (21.95~33.95) *** | 36.81  (30.77~42.85 )*** | <0.001 | 5.48  (4.60~6.36)*** |
| Percentage of left lower limb | 0(Ref.) | 11.51  (7.25~15.76)*** | 21.25  (16.98~25.51)*** | 26.20  (21.91~30.49) *** | <0.001 | 3.91  (3.29~4.54)*** |
| Percentage of right lower limb | 0(Ref.) | 11.57  (7.30~15.85)*** | 21.32  (17.03~25.61)*** | 26.31  (21.99~30.62)*** | <0.001 | 3.93  (3.30~4.56)*** |

CI, confidence interval; Q, quartile; SII, systemic immune-inflammation index.

^1^ Generalized linear regression. was used. Data was adjusted for gender, age, nation, drinking status, smoking status, income, screen time, outdoor activities, exercise income, the educational level of Parents, and Parents’ BMI.

^2^ The *P* value for the trend was assessed by assigning the median value for each quartile of SII as a continuous variable.

**P*<0.05;***P*<0.01;****P*<0.001

**Supplementary Table 3** Association between SII and body fat in the cohort study ^1^(N=1423).

|  | Quartiles of SII, ×109/L | | | | *P* for trend^2^ | Per 100×10^9^/L increasead |
| --- | --- | --- | --- | --- | --- | --- |
|  | Q1  (n=356) | Q2  (n=356) | Q3  (n=356) | Q4  (n=355) |  |  |
|  | *β*(95CI%) | | | |  | *β*(95%CI) |
| Range of SII(10^9^/L) | 40.99~282.96 | 283.20~388.23 | 388.43~526.44 | 528.00~2574.48 |  |  |
| Body fat Percentage | 0(Ref.) | 0.37  (-0.59~1.32) | 1.08  (0.12~2.03) * | 0.99  (0.03~1.94)* | 0.017 | 0.15  (0.02~0.29)* |
| Visceral fat area | 0(Ref.) | 1.26  (-0.70~3.21) | 2.18  (0.24~4.13) * | 2.55  (0.60~4.51)* | 0.006 | 0.39  (0.06~0.62)* |
| Torso | 0(Ref.) | 2.57  (-0.63~11.17) | 9.55  (1.00~18.10)* | 10.36  (1.76~18.95)* | 0.006 | 1.76  (0.53~2.98)** |
| Percentage of left upper limb | 0(Ref.) | 2.56  (-4.08~9.20) | 8.16  (1.56~14.77)* | 8.93  (2.30~15.57)** | 0.002 | 1.45  (0.50~2.40)** |
| Percentage of right upper limb | 0(Ref.) | 2.38  (-4.23~8.98) | 8.22  (1.65~14.79)* | 8.64  (2.03~15.24 )* | 0.003 | 1.42  (0.48~2.36)** |
| Percentage of left lower limb | 0(Ref.) | 2.04  (-3.81~7.89) | 7.90  (2.08~13.71)** | 7.15  (1.31~13.00) * | 0.004 | 1.14  (0.31~1.98)** |
| Percentage of right lower limb | 0(Ref.) | 2.27  (-3.61~8.15) | 7.95  (2.11~13.80)** | 7.29  (1.41~13.17)* | 0.004 | 1.16  (0.32~2.00)** |

RR, relative risk; CI, confidence interval; Q, quartile; SII, systemic immune-inflammation index.

^1^ Generalized linear regression was used. Model was adjust for gender, age, nation, drinking status, smoking status, income, screen time, outdoor activities, exercise time, the educational level of parents, and parents’ BMI.

^2^ The *P* value for the trend was assessed by assigning the median value for each quartile of SII as a continuous variable.

**P*<0.05;***P*<0.01;****P*<0.001

Figures:

**Supplementary Figure 1** Study population


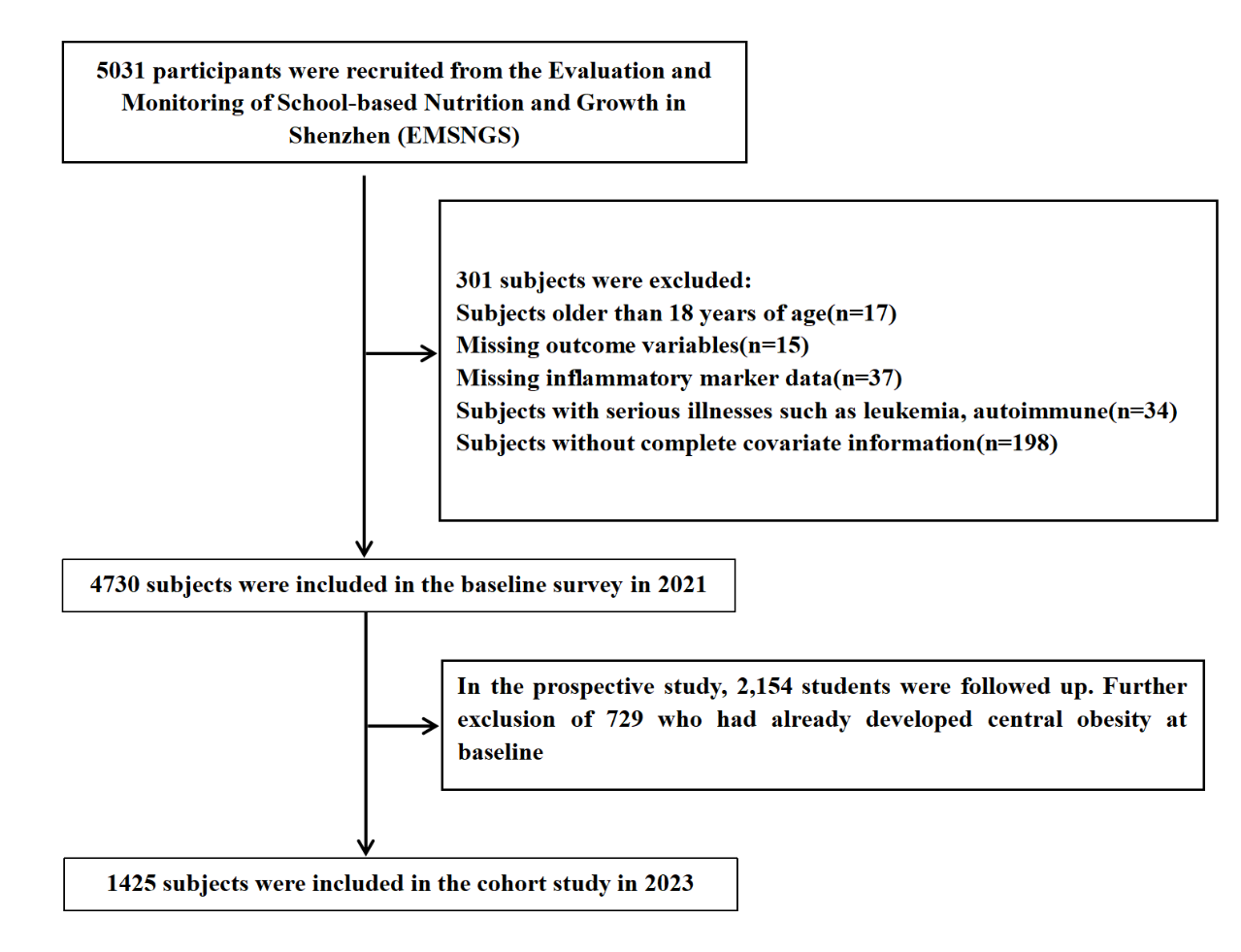


**Supplementary Figure 1** Study population
